# Pitfalls in the diagnosis and treatment of a hypertensive patient with unilateral primary aldosteronism and contralateral pheochromocytoma: a case report

**DOI:** 10.1186/s12902-023-01297-3

**Published:** 2023-02-16

**Authors:** Shotaro Miyamoto, Yuichi Yoshida, Yoshinori Ozeki, Mitsuhiro Okamoto, Koro Gotoh, Takayuki Masaki, Haruto Nishida, Hiroyuki Fujinami, Toshitaka Shin, Tsutomu Daa, Yoshiki Asayama, Hirotaka Shibata

**Affiliations:** 1 Department of Endocrinology, Metabolism, Rheumatology and Nephrology, Faculty of Medicine, Oita University, Yufu City, Oita 879-5593 Japan; 2 Department of Diagnostic Pathology, Faculty of Medicine, Oita University, Yufu City, Oita 879-5593 Japan; 3 Department of Urology, Faculty of Medicine, Oita University, Yufu City, Oita 879-5593 Japan; 4 Department of Radiology, Faculty of Medicine, Oita University, Yufu City, Oita 879-5593 Japan

**Keywords:** Primary aldosteronism, Pheochromocytoma, Secondary hypertension, Adrenal masses, Concomitant primary aldosteronism, Pheochromocytoma

## Abstract

**Background:**

Primary aldosteronism (PA) is a common cause of secondary hypertension, whereas pheochromocytoma is a rare cause of it. Thus, concomitant PA and pheochromocytoma is a very rare condition.

**Case presentation:**

A 52-year-old woman was admitted to our hospital with suspected PA based on the presence of hypertension, spontaneous hypokalemia, and a high aldosterone-to-renin ratio. She had no catecholamine excess symptoms other than hypertension. Abdominal computed tomography (CT) showed a right lipid-rich adrenal mass and a left lipid-poor adrenal mass. PA was diagnosed by the captopril challenge test. The 24-h urinary fractionated metanephrines were slightly elevated. Adrenal vein sampling (AVS) confirmed that the right adrenal gland was responsible for aldosterone hypersecretion. Medical therapy with eplerenone was started because the patient refused surgery. Five years later, she requested surgery for PA. The second AVS confirmed right unilateral hyperaldosteronism, as expected. Repeated abdominal CT showed the enlargement of the left adrenal mass. The 24-h urinary fractionated metanephrines had risen to the diagnostic level. ^123^I- metaiodobenzylguanidine (MIBG) scintigraphy showed a marked tracer uptake in the left adrenal mass with no metastatic lesion. After preoperative management with α-blockade, laparoscopic left partial adrenalectomy was performed. Immunohistochemical examination of the tumor showed chromogranin A positivity leading to the diagnosis of left pheochromocytoma.

**Conclusions:**

We report an extremely rare case of concomitant unilateral PA and contralateral pheochromocytoma. When diagnosing unilateral PA by AVS, especially in cases with a lipid-poor adrenal mass, clinicians should rule out the possibility of the presence of pheochromocytoma before proceeding to undergo unilateral adrenalectomy. Although there is no standard treatment for this rare condition, it is essential to select personalized treatment from the perspective of conserving the adrenal gland.

## Background

Hypertension is classified as essential hypertension, which has no known cause, and secondary hypertension, which results from an underlying identifiable cause [1]. Case-detection testing for secondary hypertension is crucial, particularly in young patients, because early intervention can markedly alleviate or cure hypertension. Primary aldosteronism (PA) is a common cause of secondary hypertension and accounts for 5–18% of all patients with hypertension [2–4].Pheochromocytoma is a rare cause of the condition accounting for 0.05–0.1% of all hypertensive patients [5]. Concomitant PA and pheochromocytoma is extremely rare condition; a recent case series found only 15 patients diagnosed with coexisting PA and pheochromocytoma [6]. Here, we report an extremely rare case of concomitant unilateral PA and contralateral pheochromocytoma.

## Case presentation

**Fig. 1:**
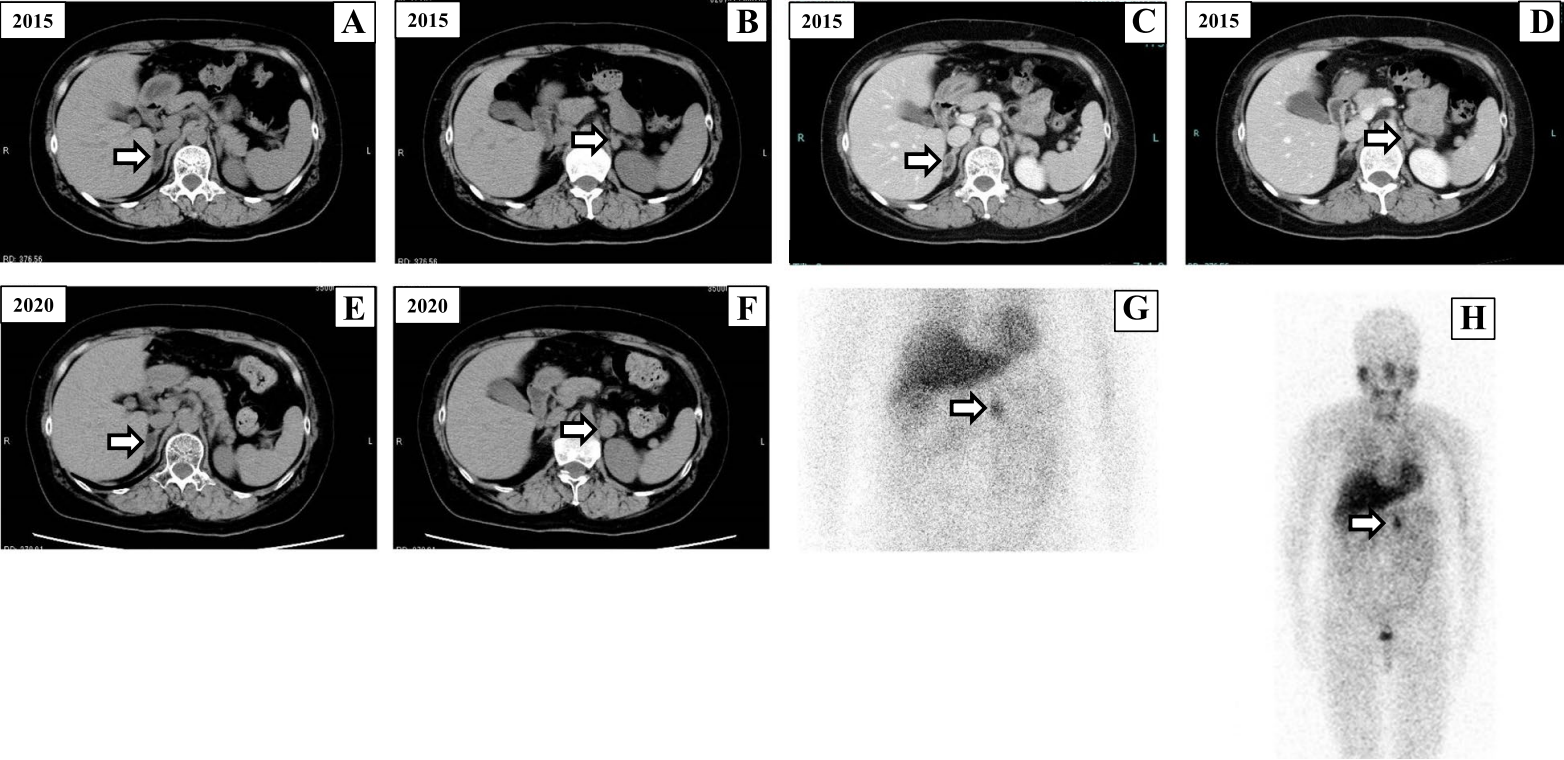
**A** and **B** Computed tomography (CT) during the initial adrenal vein sampling (AVS) revealed bilateral adrenal nodules (right:16 × 11 mm, left:13 × 9 mm) with CT attenuation values of 30–60 Hounsfield units on the left and 10 Hounsfield units on the right. **C** and **D** Contrast-enhanced CT showed contrast effect in the left adrenal nodule but not in the right. **E** and **F** CT images at the second AVS (performed 5 years after the initial AVS) showed no significant changes in the right adrenal mass and enlargement of the left adrenal nodule (26 × 13 mm). **G** and **H** ^123^I-MIBG scintigraphy showed marked uptake in the left adrenal nodule, but no metastatic lesions

**Fig. 2:**
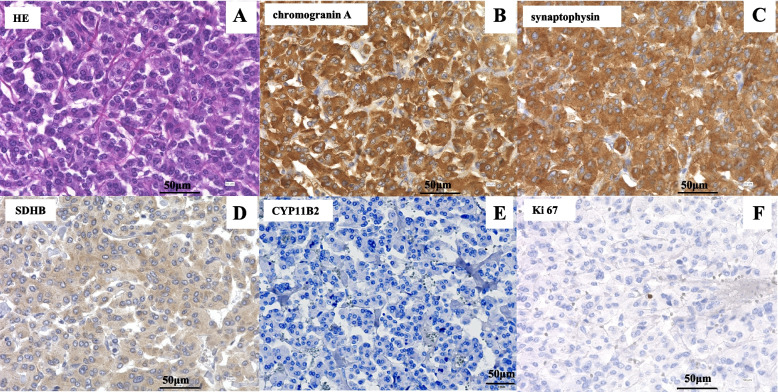
Microscopic findings of pheochromocytoma. Equipment parameters: Microscope: OLYMPUS BX51TF Objective lens: UPlanSApo X40 Cameras: OLYMPUS DP73 Acquisition software: cellSens Standard Measured resolution: 72dpi. **A-F**, Hematoxylin–eosin (HE) staining, and immunohistochemical examination of the tumor tissue. **A** HE-staining revealed that the tumor was arranged in diffuse sheets or nests, and the tumor cells were large with prominent nucleoli. **B-D** Immunohistochemical examination revealed that the tumor was positive for CgA, synaptophysin, and SDHB. **E** CYP11B2 was negative, and **F** a few Ki-67 positive cells were detected

**Fig. 3:**
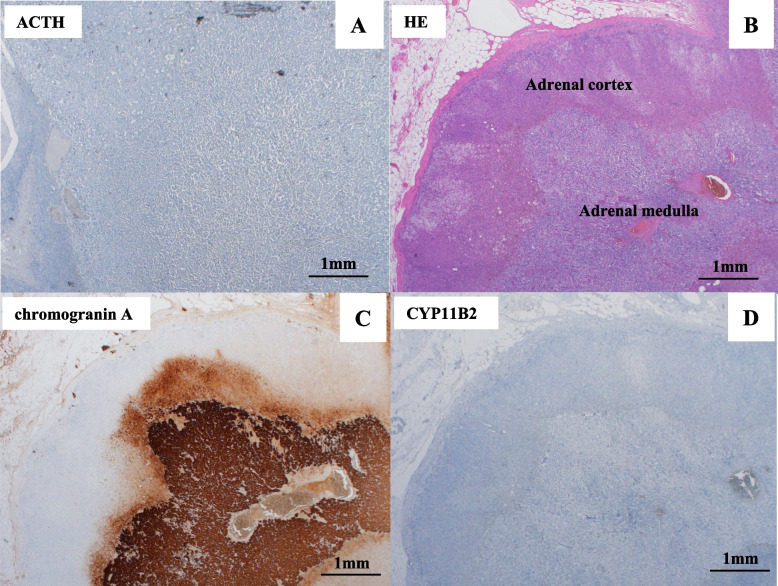
Microscopic findings of pheochromocytoma and normal adrenal gland. Equipment parameters: Microscope: OLYMPUS BX51TF Objective lens: UPlanSApo X40 Cameras: OLYMPUS DP73 Acquisition software: cellSens Standard Measured resolution: 72dpi. **A** Immunohistochemical examination of the tumor tissue. **B-D** Hematoxylin–eosin (HE) staining, and immunohistochemical examination of left adrenal gland. **A** There were no ACTH-positive cells in the tumor. **B-D** Left adrenal gland adjacent to the tumor. **B** HE-staining showed no specific change both in the adrenal cortex and the adrenal medulla. **C** CgA was positive in the adrenal medulla. **D** CYP11B2 was negative, and there were no CYP11B2 positive micronodules or diffuse hyperplasia in the adrenal cortex

A 52-year-old previously healthy woman with no family history of endocrine disease was admitted to our hospital with suspected PA based on high blood pressure (193/105 mmHg), spontaneous hypokalemia (potassium [K], 3.4 mmol/L), and a high aldosterone-to-renin ratio (ARR 71 ng/dL per ng/mL/h [plasma renin activity (PRA) 0.2 ng/mL/h and plasma aldosterone concentration (PAC) 14.2 ng/dL]). On admission, the patient showed no clinical features of Cushing syndrome, and no catecholamine excess symptoms other than hypertension, such as headache or excessive sweating. Before the initiation of antihypertensive drugs, PA was confirmed by the positive captopril challenge test (PRA: 0.2 ng/mL/h, PAC: 9.6 ng/dL, ARR: 48 [> 20]; Table 1) according to the Japan Endocrine Society Clinical Practice Guidelines for PA [7] and the Japanese Society of Hypertension Guidelines for the Management of Hypertension [8]. Abdominal computed tomography (CT) revealed bilateral adrenal masses (right, 16 × 11 mm; left, 13 × 9 mm), with baseline CT attenuation values of 30–60 Hounsfield units (HU) on the left and 10 HU on the right (Fig. 1, A and B), and contrast-enhanced CT showed a stronger contrast effect in the left adrenal mass than in the right adrenal mass (Fig. 1, C and D). Although overnight 1mg dexamethasone suppression test (DST) showed mild autonomous cortisol secretion (2.25 µg/dL [< 1.8]; Table 1), plasma level of ACTH in the early morning before DST was not suppressed (52.1 pg/mL [> 7.07]; Table 1), and the diurnal variation of serum cortisol was preserved (Table 1). Based on the Japanese Endocrine Society criteria [9], adrenal subclinical Cushing's syndrome was ruled out. The 24-h urinary catecholamines (epinephrine, 23.7 μg/day [< 41]; norepinephrine, 151.6 μg/day [< 160]; dopamine, 582.1 μg/day [< 1100]) were within normal limits, and the urinary fractionated metanephrines (metanephrine, 0.47 mg/day [< 0.19] and normetanephrine, 0.46 mg/day [< 0.33]) were slightly elevated but did not reach 3-fold of the upper limit of normal range (Table 2). Adrenal vein sampling (AVS) was performed because she requested surgical treatment at that time and the results were interpreted according to previous guidelines [7]. The findings confirmed that the right adrenal gland was responsible for aldosterone hypersecretion (post-cosyntropin stimulation lateralization ratio 24.67 [> 4], contralateral ratio 0.575 [< 1]; Table 3). She refused right adrenalectomy at the last minute, and medical therapy with the mineralocorticoid receptor (MR) antagonist, eplerenone (50 mg) was initiated; however, she abruptly discontinued treatment and the follow-up visit. Five years after the first AVS, she visited a local clinic with complaints of headache and high blood pressure (208/109 mmHg) and was administered a calcium blocker (cilnidipine 10 mg). At this time, she requested surgery for PA and was readmitted to our hospital. On admission, her blood pressure was 127/96 mmHg, and she was taking oral cilnidipine 20 mg/day. No symptoms of catecholamine excess or Cushing's signs were observed. Repeated abdominal CT showed no significant change in the right adrenal mass (16 × 11 mm); however, the left adrenal mass was enlarged to 26 × 13 mm (Fig. 1, E and F). At the surgeon's request, AVS was performed a second time to confirm PA localization. The second AVS confirmed the right unilateral hyperaldosteronism (post-cosyntropin stimulation lateralization ratio: 12.60; contralateral suppression index: 0.40) had not changed. (Table 3). Repeated 1mg DST showed mild autonomous cortisol secretion, but the diurnal variation of cortisol was preserved (11:00 pm cortisol: 4.46 µg/dL). The 24-h urinary excretion assay revealed that norepinephrine (142.3 μg/day), epinephrine (26.5 μg/day), and dopamine (644.0 μg/day) levels were normal; however, metanephrine (0.81 mg/day) and normetanephrine (0.60 mg/day) concentrations were higher than those measured 5 years earlier (Table 2). ^123^I-MIBG scintigraphy showed a tracer-avid left adrenal mass but no metastatic lesions (Fig. 1, G and H). Based on these findings, a left pheochromocytoma was diagnosed preoperatively. After the temporary discharge, we had a conference with the department of urology of our institution, and we decided to perform partial adrenalectomy for the left pheochromocytoma and medical treatment for the right unilateral PA. The patient was started on α-blocker (doxazosin, 2 mg/day), a non-steroidal MR antagonist (esaxerenone, 5 mg/day), and continued cilnidipine 20 mg/day. Two months later, she was readmitted to our hospital. After preoperative administration of saline (1 L/day) for 7 days and an α-blocker (doxazosin, 2.0 mg/day) for 2 months, laparoscopic left partial adrenalectomy was performed. No intraoperative or postoperative hemodynamic instability occurred. Histologically, the tumor cells were arranged in diffuse sheets or nests and were large with prominent nucleoli (Fig. 2A). The pheochromocytoma of the adrenal gland scaled score was 0 indicating a low grade pheochromocytoma. Immunohistochemical examination of the tumor showed that chromogranin A and synaptophysin were positive (Fig. 2, B, C), leading to the final diagnosis of left pheochromocytoma; Succinate dehydrogenase subunit B (SDHB) was positive (Fig. 2D); cytochrome P450 (CYP) 11B2 was negative, Ki 67 was weakly positive (Fig. 2, E, F), and ACTH was negative (Fig. 3A). The left adrenal gland tissue adjacent to the tumor was normal (Fig. 3, B-D). Although we could not perform genetic analysis, as consent could not be obtained from the patient, the positive immunostaining for SDHB, suggested against SDH-related paraganglioma syndromes. Doxazosin was discontinued after surgery and esaxerenone (5 mg) and cilnidipine (20 mg) were continued. One month after surgery, the patient’s blood pressure was 126/84 mmHg, and her serum K (4.27 mmol/L), PAC (47.38 ng/dL), active renin concentration (ARC) (36.57 μU/mL), and random fractionated urinary metanephrines (metanephrine 152 ng/mg Cr [29–158], normetanephrine 363 ng/mg Cr [122–500]) were normalized. One year after surgery, blood pressure and serum K levels remained well controlled. No surgical treatment for the right adrenal mass has been performed, and pheochromocytoma has not recurred.

**Table 1:** Laboratory and endocrinological data

Parameter	Values	Reference ranges
K (mmol/L)	3.67	3.6–5.0
Creatinine (mg/dL)	0.55	0.47–0.79
PAC (ng/dL)	19.7	3.6–24
PRA (ng/mL/h)	0.3	0.2–2.3
DHEA-S (µg/dL)	97	8–188
ACTH (pg/mL)	52.1	7.2–63.3
11 pm ACTH (pg/mL)	19.26	
Cortisol (µg/dL)	15.87	7.07–19.6
11 pm Cortisol (µg/dL)	3.44	< 5
Urinary Cortisol (μg/day)^a^	48.2	11.2–80.3
Urinary Aldosterone (μg/day)^a^	14.4	1.0–19.3
1 mg DST cortisol (µg/dL)	2.25	< 1.8
Captopril challenge test		
Pre-PAC (ng/dL)	18.3	3.6–24
PRA (ng/mL/h)	0.2	0.2–2.3
ARR	91.5	< 20
90 min-PAC (ng/dL)	9.6	
PRA (ng/mL/h)	0.2	
ARR	48	

*Abbreviations*: *PAC* plasma aldosterone concentration, *PRA* plasma renin activity, *DHEA-S* dehydroepiandrosterone sulfate, *ACTH* adrenocorticotrophic hormone, *DST* dexamethasone suppression test, *ARR* plasma aldosterone/renin ratio

^a^24-hour urinary testing was performed in 2020

**Table2:** Catecholamines and their metabolites changes over 5 years

	**First admission**	**After 5 years**	**Normal range**
**Plasma**			
Epinephrine (pg/mL)	30	60	< 100
Norepinephrine (pg/mL)	310	200	< 450
Dopamine (pg/mL)	< 20	< 20	< 20
**Urine**			
Epinephrine (μg/day)	23.7	26.5	< 41
Norepinephrine (μg/day)	151.6	142.3	< 160
Dopamine (μg/day)	582.1	644.0	< 1100
Metanephrine (mg/day)	0.47	0.81	< 0.19
Normetanephrine (mg/day)	0.46	0.60	< 0.33

**Table 3:** Results of Adrenal vein sampling test

	**First AVS**					**Second AVS (5 years after first AVS)**				
	aldosterone (ng/dL)	cortisol (µg/dL)	A/C	LR	CR	aldosterone (ng/dL)	cortisol (µg/dL)	A/C	LR	CR
Unstimulated										
Right adrenal vein	328.54	62.54	5.25	63.14		938.33	76.3	12.30	24.18	
Left adrenal vein	50.31	604.2	0.083		0.598	306.16	601.7	0.509		0.40
Inferior vena cava	2.33	16.78	0.139			29.63	23.4	1.265		
Cosyntropin-Stimulated										
Right adrenal vein	1903.05	803.6	2.368	24.67		635.37	698.1	9.10	12.60	
Left adrenal vein	53.05	767.8	0.096		0.575	461.9	639.6	0.722		0.40
Inferior vena cava	5.36	32.11	0.167			45.75	25.2	1.813		
Localization diagnosis			**Right**					**Right**		

*Abbreviations*: *AVS* Adrenal vein sampling test, *A* aldosterone, *C* cortisol, *LR* lateralized ratio, *CR* contralateral ratio

## Discussion and conclusions

The coexistence of PA, a common disease, and pheochromocytoma, a rare disease, is a very rare condition. To our knowledge, our case is the third report of coexisting unilateral PA confirmed by AVS and unilateral pheochromocytoma on the contralateral side [10, 11]. In our case, after the initial diagnosis of right unilateral PA by AVS, the patient refused surgery and discontinued both the medical treatment with an MR antagonist and the follow-up visit, resulting in 5 years gap. However, during this time, the left adrenal mass enlarged, and catecholamine metabolite levels increased, which enabled us to detect pheochromocytoma. The medical treatment with an MR antagonist for the right unilateral PA and the partial adrenalectomy for the left pheochromocytoma, which was different from the standard treatment for each disease, enabled the patient to preserve adrenal function and avoid replacement therapy. We report this case to highlight a potential pitfall in the diagnosis and treatment of this rare condition.

Simultaneous diagnosis of PA and pheochromocytoma is often difficult. Hypertension with spontaneous hypokalemia is a strong indication of PA; however, hypokalemia is not a common feature of PA [12, 13]. Given that the prevalence of PA in hypertensive patients is higher than previously thought, case-detection testing is recommended for hypertensive patients whose plasma aldosterone and renin have not been investigated previously. Following a positive finding, a PA diagnosis should be confirmed by at least one positive functional confirmatory test. If the patient desires surgical treatment, AVS should be performed to diagnose whether either or both adrenal glands are producing excessive aldosterone [14]. In our case, we performed AVS for subtype diagnosis in 2015 and 2020. Catheter insertion success was determined by assessment of the cortisol level in the adrenal vein relative to the inferior vena cava: before ACTH administration, cortisol levels in right and left adrenal veins were three-fold higher than in the inferior vena cava, and after ACTH administration, these were five-fold higher than in the inferior vena cava (Table 3). For localization, the aldosterone/cortisol ratio (A/C ratio) was calculated; unilateral PA was diagnosed when the A/C ratio was four-fold higher on the right side than on the left side (lateralization ratio ≥ 4) and the A/C ratio on the left side was less than in the inferior vena cava (contralateral suppression index: < 1). The findings confirmed that the right adrenal gland was responsible for aldosterone hypersecretion (post-cosyntropin stimulation lateralization ratio 24.67 (2015), 12.67 (2020) [> 4], contralateral suppression index: 0.575 (2015), 0.40 (2020) [< 1]; Table 3) [7]. Therefore, our patient had a typical unilateral PA phenotype with hypertension, high plasma aldosterone, suppressed PRA, and subtype testing using AVS revealed the right unilateral PA.

In contrast, pheochromocytoma is characterized by catecholamine excess symptoms including hypertension, headache, palpitations, and sweating. However, an increasing number of small pheochromocytomas that are asymptomatic and no biochemical evidence of catecholamine excess (pre-biochemical) have been reported due to increased use of CT for abdominal imaging or family screening [15]. Unlike PA, pheochromocytomas are often not biologically active until they have a substantial size (> 1.5 cm in diameter). pheochromocytoma is diagnosed based on the presence of elevated catecholamine and/or their metabolite levels and imaging findings of a tumor [16]. Lipid-poor and vascular adrenal masses with higher CT attenuation (> 10HU) than adrenal adenomas should be considered suspicious for pheochromocytoma and small adrenal cortical cancer. In our case, both adrenal nodules (right:16 × 11 mm, left:13 × 9 mm) were not large enough in tumor size at the time of first discovery. Still, the right lipid-rich adrenal nodule was a florid aldosterone-producing tumor with hypertension, low K, and high aldosterone. On the other hand, the left lipid-poor adrenal nodule was a nonfunctional and pre-biochemical pheochromocytoma. This difference posed a significant challenge for simultaneous diagnosis. However, since the urinary fractionated metanephrines levels were already between the upper limit of the normal range and the diagnostic level and the CT attenuation of the left adrenal nodule was more than 30 HU at the first admission, we should have performed an additional investigation including a clonidine suppression test for adrenal nodules with possible pheochromocytoma [16, 17]. Instead, we decided to do a close follow-up, but unfortunately, she interrupted it. Finally, the growing size of the left adrenal tumor (Fig. 1) and the elevation of 24-h urinary fractionated metanephrines to the diagnostic level (Table 2) during this period enabled us to diagnose the left pheochromocytoma. Our case demonstrated the importance of a long-term follow-up by confirming the elevation of catecholamine metabolites and the growth of the tumor to diagnose an initially small, less biochemically active pheochromocytoma.

When we diagnose unilateral PA by AVS, it is essential to exclude the coexistence of pheochromocytoma before adrenalectomy for two reasons. First, in cases of coexisting pheochromocytoma, the preoperative α-adrenergic blockade is necessary to prevent a hypertensive crisis caused by the rapid release of catecholamines from the tumor due to intraoperative stimulation [18]. In our case, if unilateral right adrenalectomy had been performed without confirming the coexistence of left pheochromocytoma, it could have caused an intraoperative hypertensive event resulting in cardiovascular complications.

Second, when unilateral PA and pheochromocytoma are present on the contralateral side, as in our case, personalized treatment should be considered to prevent permanent hypocortisolism after complete bilateral adrenalectomy. Medical treatment with an MR antagonist is the standard treatment for bilateral PA because it improves hypertension and other deleterious cardiovascular effects [19], whereas complete adrenalectomy is recommended for unilateral PA because of lifetime costs and effectiveness in improving hypertension and hypokalemia [20, 21]. The standard treatment for pheochromocytoma is complete adrenalectomy; however, partial adrenalectomy is recommended for selected patients with prior contralateral adrenalectomy or bilateral pheochromocytomas [22]. In the present case, we treated the right unilateral PA with an MR antagonist and performed partial adrenalectomy to treat the left pheochromocytoma, which differs from the standard procedures for each disease. However, our personalized treatment reduced the possibility of permanent adrenal corticosteroid replacement therapy if right complete adrenalectomy for unilateral PA should be necessary in the future. Therefore, we recommend that in cases of unilateral PA with suspected contralateral pheochromocytoma, unilateral adrenalectomy should not be used to treat unilateral PA until the coexistence of pheochromocytoma has been completely ruled out. If unilateral PA and contralateral pheochromocytoma coexist, we should treat unilateral PA with an MR antagonist to preserve an adrenal gland and perform surgical resection for the contralateral pheochromocytoma. Of course, should blood pressure increase and serum K levels become suboptimal in the future, right complete adrenalectomy should be considered. Moreover, partial adrenalectomy for pheochromocytoma means a higher risk of recurrence than complete adrenalectomy. So, long-term follow-up with biochemical and imaging findings to assess PA disease activity and the recurrence of pheochromocytoma is of paramount importance for this case.

Last, the mild autonomous secretion of cortisol after 1mg DST in both 2015 and 2020 should be discussed. First, the patient showed no clinical feature of Cushing’s syndrome, which ruled out Cushing’s syndrome. In addition, the diurnal variation of serum cortisol was not disappeared, which ruled out subclinical Cushing's syndrome according to the guidelines of the Japan Endocrine Society [9]. We also suspected the existence of ACTH-producing pheochromocytoma because both AVS tests showed much higher cortisol level in left adrenal vein than in the right adrenal vein and ACTH- stimulation didn’t adequately increase the cortisol levels in the left adrenal vein (Table 3). However, there were no ACTH-positive cells in the tumor tissue (Fig. 3A), which ruled out ACTH-producing pheochromocytoma. Based on these findings, we concluded that the autonomous secretion of cortisol was not so strong that caused Cushing’s or subclinical Cushing’s syndrome. However, since it is possible that the right aldosterone-producing tumor is also producing cortisol, the patient should be closely monitored for the complication of subclinical Cushing's or Cushing's syndrome in the future.

In conclusion, when clinicians encounter unilateral PA with a lipid-poor, vascular adrenal mass, the coexistence of pheochromocytoma should be excluded. Even if the tumor is small and is in the pre-biochemical phase, it should be closely monitored for tumor growth and an associated increase in catecholamine metabolite levels, which should help diagnose pheochromocytoma. Although the standard treatment for unilateral PA is unilateral adrenalectomy, in cases of unilateral PA with contralateral pheochromocytoma, we suggest trying the medical treatment with an MR antagonist first to preserve adrenal function.

## Data Availability

Data sharing is not applicable to this article as this article is a case report, and there is a risk that the patient's personal information may be leaked in the process of sharing the data.

## Abbreviations

PA: Primary aldosteronism
CT: Computed tomography
HU: Hounsfield units
AVS: Adrenal vein sampling
MR: Mineralocorticoid receptor
ARR: Aldosterone-to-renin ratio
PAC: Plasma aldosterone concentration
PRA: Plasma renin activity
ARC: Active renin concentration
ACTH: Adrenocorticotropic hormone
A/C: Aldosterone/cortisol
FDG-PET: Fluorodeoxyglucose-position emission tomography
MIBG: Metaiodobenzylguanidine
CgA: Chromogranin A
HE: hematoxylin–eosin
SDHB: succinate dehydrogenase subunit B
CYP: Cytochrome P450
